# Changes in JC Virus-Specific T Cell Responses during Natalizumab Treatment and in Natalizumab-Associated Progressive Multifocal Leukoencephalopathy

**DOI:** 10.1371/journal.ppat.1003014

**Published:** 2012-11-08

**Authors:** Molly R. Perkins, Caroline Ryschkewitsch, Julia C. Liebner, Maria Chiara G. Monaco, Danielle Himelfarb, Sara Ireland, Annelys Roque, Heather L. Edward, Peter N. Jensen, Gina Remington, Thomas Abraham, Jaspreet Abraham, Benjamin Greenberg, Charles Kaufman, Chris LaGanke, Nancy L. Monson, Xiaoning Xu, Elliot Frohman, Eugene O. Major, Daniel C. Douek

**Affiliations:** 1 Human Immunology Section, Vaccine Research Center, National Institute of Allergy and Infectious Disease, National Institutes of Health, Bethesda, Maryland, United States of America; 2 MRC Human Immunology Unit, Weatherall Institute of Molecular Medicine, University of Oxford, Oxford, United Kingdom; 3 Laboratory of Molecular Medicine and Neuroscience, National Institute of Neurological Disorders and Stroke, National Institutes of Health, Bethesda, Maryland, United States of America; 4 Department of Neurology, University of Texas Southwestern Medical Center, Dallas, Texas, United States of America; 5 Louisiana Neurologic Consultants. Baton Rouge, Louisiana, United States of America; 6 North Central Neurology, Cullman, Alabama, United States of America; University of Michigan, United States of America

## Abstract

Progressive multifocal leukoencephalopathy (PML) induced by JC virus (JCV) is a risk for natalizumab-treated multiple sclerosis (MS) patients. Here we characterize the JCV-specific T cell responses in healthy donors and natalizumab-treated MS patients to reveal functional differences that may account for the development of natalizumab-associated PML. CD4 and CD8 T cell responses specific for all JCV proteins were readily identified in MS patients and healthy volunteers. The magnitude and quality of responses to JCV and cytomegalovirus (CMV) did not change from baseline through several months of natalizumab therapy. However, the frequency of T cells producing IL-10 upon mitogenic stimulation transiently increased after the first dose. In addition, MS patients with natalizumab-associated PML were distinguished from all other subjects in that they either had no detectable JCV-specific T cell response or had JCV-specific CD4 T cell responses uniquely dominated by IL-10 production. Additionally, IL-10 levels were higher in the CSF of individuals with recently diagnosed PML. Thus, natalizumab-treated MS patients with PML have absent or aberrant JCV-specific T cell responses compared with non-PML patients, and changes in T cell-mediated control of JCV replication may be a risk factor for developing PML. Our data suggest further approaches to improved monitoring, treatment and prevention of PML in natalizumab-treated patients.

## Author Summary

Progressive multifocal leukoencephalopathy (PML) is a complication of treatment with natalizumab in patients with multiple sclerosis (MS) and Crohn's disease. PML results from a failure of the immune system to control replication of JC virus (JCV) in the brain. We studied the T cell responses of 8 patients with MS who were starting treatment with natalizumab, 10 healthy volunteers, and 4 patients with natalizumab-associated PML. The magnitude and quality of JCV-specific immune responses remained unchanged after starting natalizumab. However, applying the same methods and antigens, we found that immune responses in the individuals who developed PML differed from those in the MS patients and healthy volunteers. In the four patients with PML from whom the laboratory had identified JCV DNA in the cerebrospinal fluid (CSF), two had no measurable T cell response to JCV and two had T cells that produced IL-10, an anti-inflammatory mediator. Furthermore, we studied the CSF of 10 patients with natalizumab-associated PML and 10 patients on natalizumab who had similar symptoms but did not have PML. We found that IL-10 was detectable in the CSF of half of the individuals with PML but none of the control group. These findings shed light on the mechanisms that lead to PML in a subset of patients treated with natalizumab and have implications for therapeutic and preventative measures.

## Introduction

Progressive Multifocal Leukoencephalopathy (PML) is a demyelinating disease of the central nervous system caused by JC virus (JCV) [1]. PML occurs most often in the setting of immunodeficiency, such as AIDS, leukemia, organ transplantation and idiopathic CD4 lymphopenia. However, cases of PML have recently been reported after immunotherapy with monoclonal antibodies, including approximately 260 cases in MS patients treated with natalizumab (Tysabri) as of July 30, 2012. The risk of PML increases with the number of natalizumab doses administered with highest incidence after 24 [2]–[4]. The 2.1/1000 overall risk of PML [2] is a major consideration in the decision to treat with natalizumab. The mechanism underlying JCV reactivation in natalizumab-treated individuals with MS is actively being investigated, but may involve both reduced immune surveillance of the central nervous system due to attenuated extravasation of leukocytes out of the bloodstream and into tissues [5]–[7], and also latent viral infection in CD34^+^ cells that migrate from the bone marrow to the peripheral circulation [8]. In this context, characterization of the effects of natalizumab on T cell immune control of JCV replication might shed light on the host and viral factors that determine the risk for the development of PML.

Previous studies have measured CD4 and CD8 T cell responses to JCV in individuals with PML who had not received natalizumab therapy [9]–[14]. CD8 T cell responses to particular JCV epitopes were found to be associated with longer survival times after early PML in HIV-positive subjects [15], and a recent study showed that CD4 and CD8 T cell responses to JCV were more likely to be detected in PML survivors than in PML progressors [16]. Most studies have focused solely on the magnitude of JCV-specific T cell responses but the quality of the response also may be important [17]. In one study, HIV-related PML was associated with a unique increase in JCV-specific interleukin 10 (IL-10) production by bulk cultures of peripheral blood mononuclear cells (PBMC), compared with non-HIV PML samples [18]. Little is known about the quality of the JCV-specific response in natalizumab-treated individuals. It has been reported that after natalizumab treatment, the levels of mRNA for the cytokines interferon gamma (IFNγ) and IL-10 change, with IFNγ levels increasing in PBMC and decreasing in CSF cells and IL-10 levels increasing in CSF cells [19]. However, as these levels were measured in bulk cultures from each compartment, it is not possible to determine whether this finding simply reflects the altered frequencies of different cell types in the blood and CSF that are known to occur with treatment [7], [20]–[22].

Two recent studies yielded disparate results when examining longitudinal T cell responses to JCV in individuals with MS after natalizumab treatment [23], [24]. Notably, these studies measured JCV-specific T cell responses directed against the VP1 protein by interferon gamma production. We hypothesized that immune control of JCV viremia in natalizumab-treated individuals might depend on T cells directed at other portions of the virus and/or on the range of the cytokines produced. To investigate the JCV-specific T cell response in greater depth, we performed a detailed characterization of the functional T cell response to the entire JC virus proteome longitudinally in individuals initiating treatment with natalizumab, and in individuals who had developed PML after treatment with natalizumab.

## Results

### Detection of JCV-specific T cell responses

We characterized the T cell immune response to JCV in eight patients with MS initiating natalizumab and ten healthy volunteers. As T cell responses have not been described to JCV proteins other than VP1, we sought to determine whether either CD4 or CD8 T cells were directed against the other portions of the virus. We stimulated PBMC with 5 pools of peptides covering the entire JCV proteome: large T antigen, small t antigen, VP1, VP2 and agnoprotein. The peptides were 15mers overlapping by 11 amino acids to optimize coverage of both CD4 and CD8 T cell epitopes, and included additional peptides to cover JCV sequence variants identified in the literature (as described in the materials and methods). Responses were measured by intracellular cytokine staining (ICS) and polychromatic flow cytometry for cytokines associated with effective control of viral infections: IFNγ, tumor necrosis factor (TNF) and interleukin 2 (IL-2), and for a cytokine associated with poor control of viral infections [25]–[27], interleukin 10 (IL-10).

**Figure 1:**
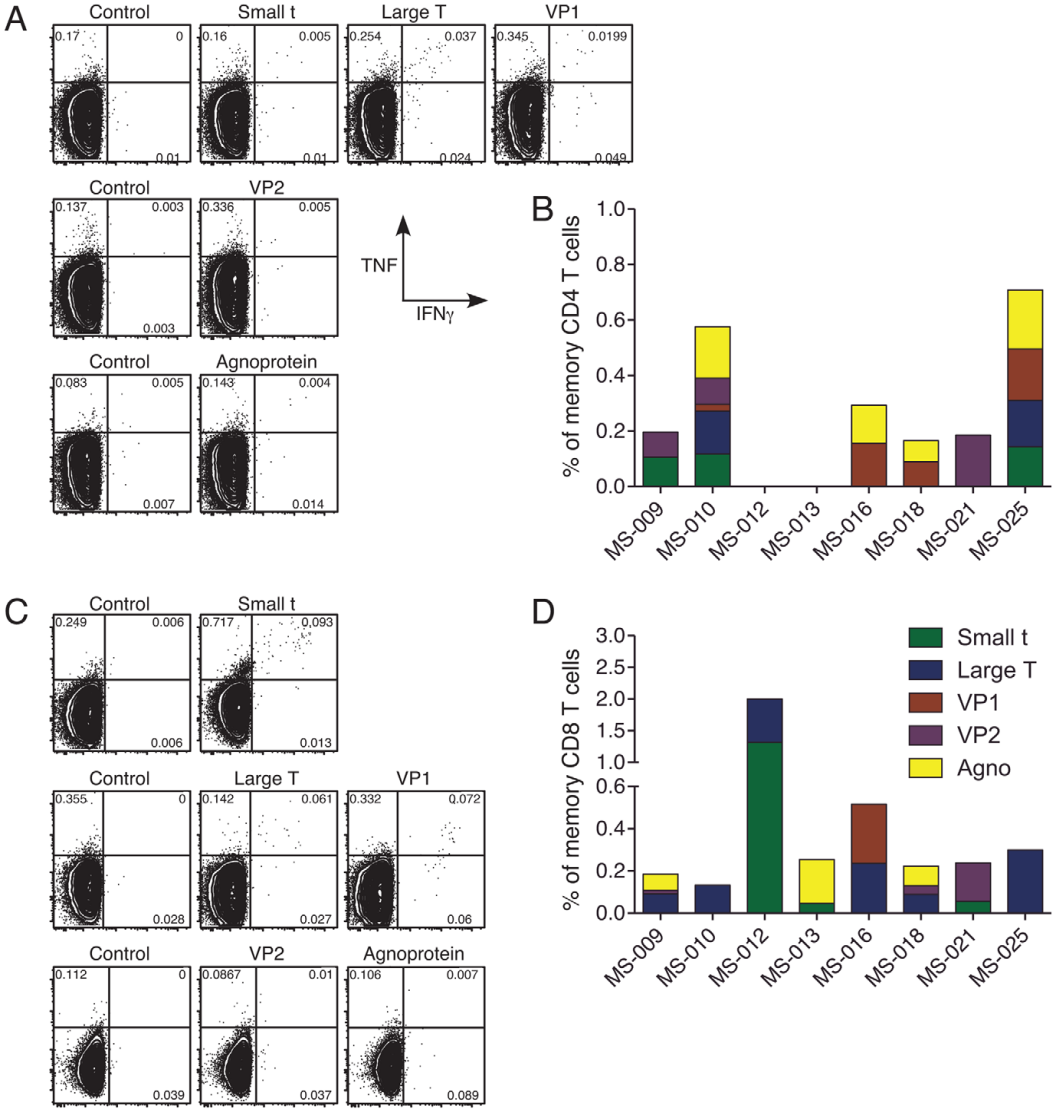
T cell responses to JC virus target each viral protein. PBMC from individuals with MS treated with natalalizumab who did not have PML were stimulated with JCV peptide pools or costimulatory molecules alone (negative control) for 6 hours. Panel A shows memory CD4 T cells from 3 samples; Panel C shows memory CD8 T cells from 3 samples. The fluorescence intensity of IFNγ and TNF are shown on the X and Y-axes, respectively. Panels B and D show baseline pre-treatment responses from all eight longitudinal subjects with MS, with the background-subtracted magnitude of the response to each JCV protein depicted by colored bars. Responses were measured by production of any combination of IFNγ, TNF and IL-2, using Boolean gates and then background subtracting from each Boolean population.

**Figure 2:**
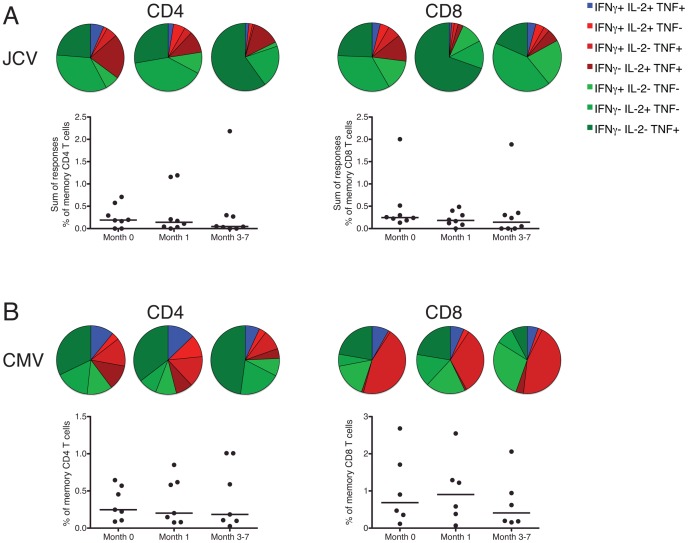
Magnitude and functional profile of JCV and CMV-specific T cells do not change upon treatment. PBMC from individuals with MS treated with natalalizumab who did not have PML were stimulated with JCV peptide pools, CMV pp65 peptide pool or costimulatory molecules alone (negative control) for 6 hours. A response was considered positive if the frequency of memory T cells producing IFNγ, TNF or IL-2 was higher in the peptide-stimulated cells than in those stimulated with costimulatory molecules alone. Response size was calculated by measuring the frequency of cells producing each Boolean combination of cytokines, and subtracting the frequency of these cells in the negative control. Summing the background-subtracted Boolean subsets gave the total frequency of cytokine-producing memory T cells specific for the peptide pool. The total response to JCV (Panel A) was calculated by summing the frequency of cells specific for each of the 5 JCV peptide pools. The functional profile of the response is shown in the pie charts above, with the blue slice representing the proportion of responding cells that produce all 3 cytokines, the red slices representing the proportion of cells that produce a combination of 2 cytokines, and the green slices representing the proportion of cells that produce only 1 cytokine. Panel B shows the frequency of CD4 (left) and CD8 (right) memory T cells responding to the CMV pp65 peptide pool.

Both CD4 and CD8 memory T cell responses were readily measured *ex vivo* from cryopreserved PBMC samples. Responses were observed in all MS patients that variously targeted each of the JCV proteins: large T antigen, small t antigen, VP1, VP2 and agnoprotein (Figure 1). Notably, different viral proteins were immunodominant in different individuals and most individuals targeted more than one protein (Figure 1). Both CD4 and CD8 T cells specific for JCV were observed in most subjects (Figure 1). None of the responding T cells produced IL-10, but each produced one or more of the other three cytokines, with a significant fraction expressing two or three cytokines (Figure 1, Figure 2A and data not shown). The specificity, magnitude and functional profile of these responses varied among individuals. Similar JCV-specific T cell responses were observed in all ten healthy subjects (Figure S1).

### Longitudinal antiviral immune responses in natalizumab-treated individuals

To determine the impact of natalizumab after short-term treatment, when risk of PML is low, we compared T cell responses to JCV and cytomegalovirus (CMV) before and after the initiation of natalizumab. The eight individuals with MS were examined at baseline (month 0) before natalizumab therapy, one month after the first infusion of natalizumab (month 1), and at the latest timepoint available (after 3–7 monthly infusions).

The total CD4 and CD8 T cell responses to JCV, calculated as the sum of the memory response to each of the five JCV peptide pools, did not change significantly at different timepoints (Figure 2A). There was also no significant difference in the magnitude of the responses to each individual peptide pool (data not shown). The functional profile of the CD4 and CD8 T cell responses to JCV, defined by production of a combination of IFNγ, TNF and IL-2, did not vary significantly over time (Figure 2A). Although the relative sizes of the slices of the pie charts representing different combinations of cytokines produced by the T cells varied across timepoints, these apparent differences were not statistically significant as determined by the chi squared and permutation tests which were used to calculate *P* values [28]. We therefore observed no change in the magnitude, quality, or targeting of the JCV-specific immune response early after initiation of natalizumab treatment.

CMV was chosen as a control antigen for JCV because it is a prevalent DNA virus that, like JCV, is neurotropic and establishes latent infection. The CMV pp65-specific CD4 and CD8 memory T cell responses did not change in magnitude or functional profile between the three timepoints (Figure 2B). None of the T cells directed against JCV or CMV produced IL-10 at any timepoint. These findings suggest that short-term natalizumab therapy does not alter these antiviral immune responses.

### Cellular immune response to mitogenic stimulation in natalizumab-treated individuals

**Figure 3:**
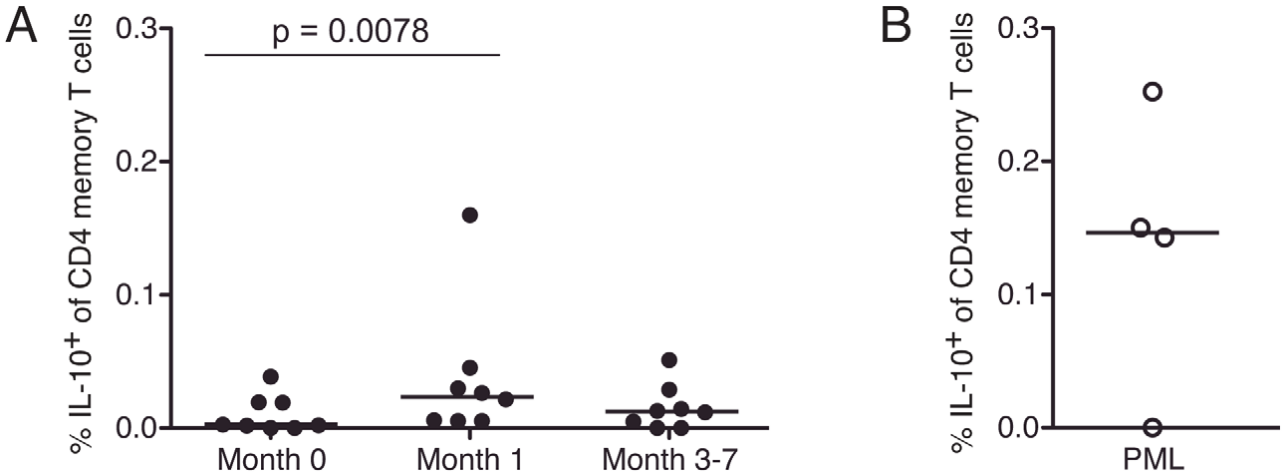
Transient increase in IL-10-producing cells after natalizumab initiation and high frequency in individuals with PML. PBMC from all studied MS patients were stimulated with SEB. Background-subtracted frequency of memory CD4 T cells producing IL-10 is shown. *P* value from month 0 to month 1 is the result of Wilcoxon matched-pairs rank sum test. Panel A shows longitudinal samples from MS patients treated with natalizumab who did not have PML, and Panel B shows samples from 4 individuals with natalizumab-associated PML.

To gauge the impact of natalizumab on the overall memory T cell response to stimulation we measured cytokine production after stimulation with the mitogen SEB. No difference was observed in the magnitude or profile of IFNγ, TNF and IL-2 produced at the different time points in response to SEB stimulation (data not shown). We also measured IL-10 production because this regulatory cytokine is associated with poor control of persistent viral infections [25]–[27]. Upon mitogenic stimulation with SEB, a small number of CD4 T cells produced IL-10. The frequency of these cells increased after one dose of natalizumab, although this effect was not sustained at later timepoints (Figure 3A). This increase in the frequency of IL-10-producing memory CD4 T cells is unlikely to result from a change in the frequency of the parent population (total memory CD4 T cells) as the frequency of these cells did not change across timepoints (data not shown). The increased frequency of T cells that produce IL-10 after one dose of natalizumab may indicate its potential to skew the functional profiles of T cells.

In order to determine whether MS patients with natalizumab-associated PML had similar T cell responses, we also examined the response to mitogenic stimulation in T cells from four patients whose CSF samples were previously tested in our laboratory for JCV DNA. One of these patients, PML-4, was one of the first cases diagnosed in 2005, who was followed for more than 5 years and never cleared JCV from the brain [29]. Importantly, in three out of the four subjects (PML-1, PML-3 and PML-4), a high frequency of CD4 T cells produced IL-10 in response to mitogenic stimulation with SEB, despite the discontinuation of natalizumab in these individuals (Figure 3B). T cells from subject PML-2 did not produce IL-10 in response to JCV, CMV or SEB stimulation and this finding was confirmed in a second sample from PML-2 (data not shown). The observation that the frequency of IL-10 producing memory CD4 T cells was high in subjects with natalizumab-associated PML raises the intriguing possibility that the transient increase in these cells after treatment initiation might indicate a potential role for natalizumab in skewing the immune response toward a cytokine associated with PML.

### Antiviral immune responses in individuals with natalizumab-associated PML

Approximately 2.1/1000 MS patients treated with natalizumab develop PML [2]. We hypothesized that such individuals might have an aberrant T cell response to JCV. Given our findings that JCV-specific T cells may target many of the JCV antigens and are varied in functional profile among individuals, we sought to identify whether there were differences in the antiviral immune responses of individuals who developed PML after treatment with natalizumab. We therefore characterized T cell responses in four individuals with natalizumab-associated PML by measuring JCV-specific production of IFNγ, TNF, IL-2 and IL-10.

**Figure 4:**
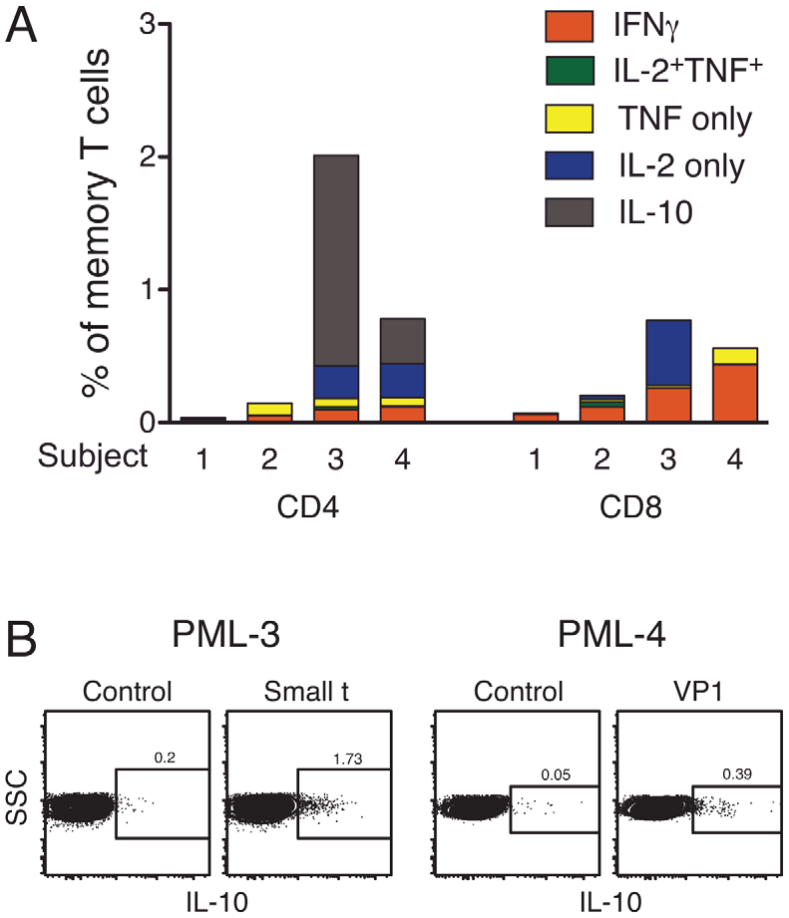
JCV-specific T cell responses in subjects with PML. Panel A shows the total response to JCV. For each of 4 subjects with PML, the summed frequency of memory CD4 (left) and CD8 (right) T cells producing IFNγ, TNF, IL-2 or IL-10 (indicated by colors) in response to all JCV peptides is shown. Red bars indicate frequency of cells producing IFNγ, including those that produce any combination of IL-2 and TNF in addition to IFNγ. The responses for subjects PML-1 and PML-2 are not significantly above background in these samples. Panel B shows the JCV-specific IL-10 response in subjects PML-3 and PML-4. Subjects PML-1, 2, 3 and 4 were sampled 2 weeks, 2 months, 4 months and 5 years, respectively, after diagnosis with PML.

In two subjects, PML-1 and PML-2, no JCV-specific CD4 or CD8 T cell responses were observed that were significantly above background (Figure 4A). These were the only subjects in whom no JCV-specific T cell responses were observed, as all the MS patients and healthy volunteers had detectable responses. The sample from PML-1 was taken 2 weeks after diagnosis with PML, and the sample from PML-2 was taken 2 months after diagnosis. Both subjects were still being followed 2 years after diagnosis.

In PML-3 and PML-4, the JCV-specific T cell response had a markedly different functional profile than was observed in any of the non-PML or healthy subjects. These subjects, who were diagnosed with PML 4 months and 5 years prior to sampling, respectively, had JCV-specific CD4 T cells that produced IL-10 (Figure 4B). The specificity of the IL-10 response for PML-4 was confirmed by double staining with the same antibody conjugated to two different fluorophores (Figure S2). The antigen specificity of the subjects' responses differed. In subject PML-3, 1.5% of the CD4 memory T cells produced IL-10 and were specific for the small t antigen (Figure 4A, grey bar), while the sum of the IFNγ responses to all of the JCV peptide pools was 0.10% (Figure 4A, red bar). The total IFNγ-producing memory CD8 T cell response was 0.26%. This subject died 1 month after sampling. In subject PML-4, 0.34% of the CD4 memory T cells produced IL-10 and were specific for VP1, while the sum of the IFNγ responses to all of the JCV peptide pools was 0.12% of CD4 memory cells. The total IFNγ-producing CD8 memory T cell response was 0.44%. Subject PML-4 died 1 year after sampling. Importantly, these were the only IL-10-producing virus-specific T cell responses we observed among all the subjects examined in this study. Thus, in contrast to non-PML MS patients or healthy volunteers, individuals who developed PML had JCV-specific T cell responses that were either uniquely dominated by IL-10 producing cells or were undetectable.

### CSF cytokines in natalizumab-associated PML

**Figure 5:**
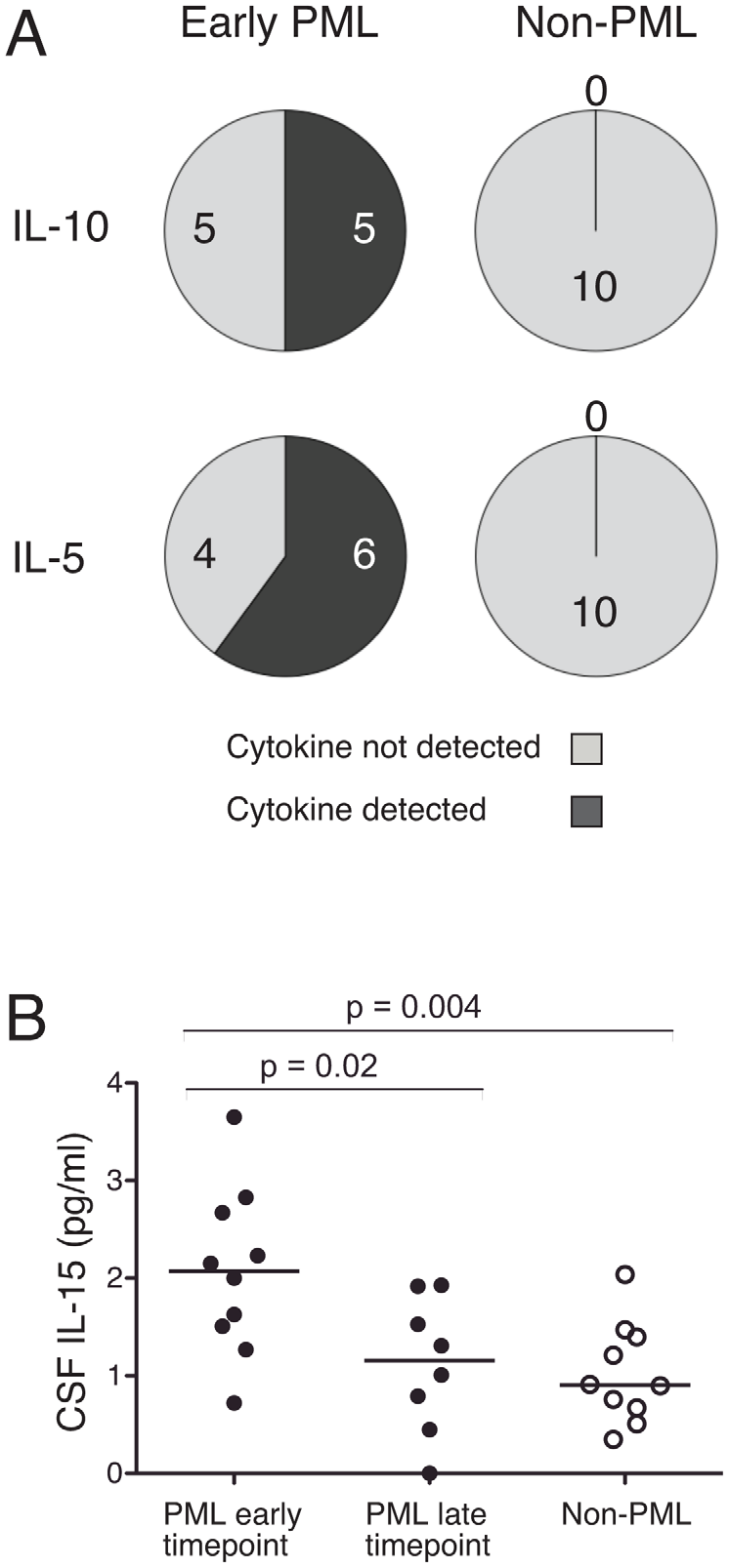
CSF cytokine levels that vary between subjects with and without natalizumab-associated PML. Diagnostic CSF samples taken from subjects with natalizumab-associated PML early in disease course were compared to samples from subjects who had suspected natalizumab-associated PML but tested negative for JCV DNA. Panel A shows the proportion of CSF samples with detectable IL-10 in diagnostic samples from subjects in whom natalizumab-associated PML was confirmed or ruled out, and the proportion of CSF samples in each group with IL-5 levels above the lower limit of quantification (1.1 pg/mL). *P* values were calculated by Fisher's exact test. Panel B shows IL-15 levels in the CSF samples. Comparison of the non-PML and early time point PML samples was by Mann-Whitney test. For 8/10 subjects with PML, a later time point was available. *P* values between paired longitudinal samples were calculated by Wilcoxon matched-pairs signed rank test.

As JCV-specific T cells in the blood of the four individuals with PML were either absent or of unusual functionality, we next examined the cytokine profile within CSF samples from 10 individuals with natalizumab-associated PML, including subjects PML 1–4, at the time of initial diagnosis. In addition, a second CSF sample from a later time point was available for 8 of these subjects. The samples were tested for the presence of 27 cytokines and other markers and were compared to diagnostic CSF samples from 10 individuals who had suspected natalizumab-associated PML which was subsequently ruled out by a negative PCR for JCV. Twelve of the molecules were undetectable in the vast majority of CSF samples, including IL-1β, IL-2, IL-4, IL-12p70, IL-17, Eotaxin, FGF basic, GM-CSF, IFNγ, MIP1α, RANTES, and TNF. An additional 12 molecules, IL-1ra, IL-6, IL-7, IL-8, IL-9, IL-13, G-CSF, IP-10, MCP-1, PDGF-β, MIP1β and VEGF, were measured in the CSF samples but did not vary in PML and non-PML samples. Although the assay was not sensitive enough for accurate quantification of low levels of IL-10, it could readily distinguish whether IL-10 was detectable above background. Thus, we found that 50% of the PML CSF samples had detectable IL-10, while none of the non-PML samples had detectable IL-10 (*P* = 0.0325, Figure 5A). Similarly, for IL-5, 60% of the PML samples had IL-5 levels above the limit of quantification, while none of the non-PML samples had IL-5 levels that could be quantitatively measured (*P* = 0.0108, Figure 5A). Thus, higher levels of IL-10 and IL-5 can be detected in the CSF of individuals with natalizumab-associated PML. Finally, in the samples from early in PML disease, levels of IL-15 in the CSF were significantly higher than in later samples from the same subjects (*P* = 0.02), or in the samples from non-PML subjects (*P* = 0.004). These results reveal a PML-specific CSF cytokine profile that may reflect the altered cytokine profile we observed in JCV-specific T cells in the blood.

## Discussion

The factors that lead to the development of PML in individuals treated with natalizumab need to be investigated in more detail, particularly immune responses to the virus. Consequently, we investigated the T cell immune response to the entire JC virus proteome longitudinally in subjects with MS who were initiating therapy with natalizumab and in subjects who had natalizumab-associated PML. The principal findings were: 1) T cell responses were identified against all JC virus proteins and could be measured *ex vivo* in the peripheral blood of individuals treated with natalizumab and healthy subjects; 2) the magnitude and quality of T cell responses to JCV and CMV did not change from baseline through the first several months of natalizumab treatment; 3) the frequency of T cells producing the cytokine IL-10 in response to mitogenic stimulation temporarily increased after the first dose of natalizumab; 4) individuals with PML either made no detectable T cell responses to JCV or had JCV-specific CD4 T cell responses uniquely dominated by IL-10 production rather than IFNγ; and 5) individuals shortly after PML diagnosis had higher levels of IL-10, IL-5 and IL-15 in the CSF.

Whether JCV-specific T cell responses can be reliably measured *ex vivo* [30], [31] and the effect of natalizumab on these responses [23], [24] have both been the subject of some debate. Using *ex vivo* stimulation with overlapping peptides, we readily detected CD4 and CD8 T cell responses to JCV by multiparameter ICS in healthy subjects, subjects with MS and in some subjects with PML. This approach could be useful in monitoring JCV-specific T cell function in natalizumab-treated individuals in the context of a vaccine or a risk stratification protocol. Our findings that JCV-specific T cells are directed against each of the viral proteins and that the specificity and immunodominance of the response varies among individuals strongly suggest that measuring responses to all viral proteins rather than VP1 alone is essential to obtaining a complete picture of JCV-specific immunity. Our results also suggest that measuring multiple cytokines rather than IFNγ alone allows for the identification of associations that would otherwise be missed, and in particular highlight the potential importance of IL-10 in evaluating T cell responses to JCV. In our longitudinal study we found no difference in either the magnitude or functional profile of the total JCV-specific T cell response during short-term natalizumab treatment. Although the sample size of our study was limited in power to detect longitudinal differences, the fact that no difference was observed in any of the cytokines measured, or in their relative contribution to the response at different time points, suggests that JCV-specific T cell responses are not altered simply by the initiation of natalizumab.

However, in the subjects with PML, JCV-specific CD4 T cell responses were either undetectable or uniquely dominated by IL-10. Importantly, it has been shown that IL-10 is detrimental to the clearance of lymphocytic choriomeningitis virus (LCMV) infection because of its inhibitory effect on virus-specific memory CD4 T cells [25], [26]. Furthermore, vaccines which stimulate CD4 T cell IL-10 production can limit the elicitation of protective polyfunctional CD4 T cells [32]. Thus, the production of IL-10 but not IFNγ, TNF or IL-2 by JCV-specific CD4 T cells may interfere with antiviral activity to the detriment of control of JCV replication, either locally in the CNS or in peripheral tissues, and may consequently be causative of PML. Another interpretation is that IL-10 production, which was not detected until >4 months after the subjects had developed PML, is a later-stage response to the inflammation often associated with PML (PML-IRIS). This sample size does not allow us definitively to link the time since diagnosis to the presence of an IL-10 response. However, our finding that IL-10 was detectable in 50% of early CSF samples suggests that IL-10 production may occur early in natalizumab-associated PML disease. Importantly, these two interpretations are not mutually exclusive. Although the role of IL-10 in PML associated with other conditions has not been thoroughly characterized, one study found increased JCV-specific IL-10 production by total PBMC from HIV^+^ PML cases, but not from non-HIV PML cases [18]. This result suggests that IL-10 may be associated with PML resulting from causes other than natalizumab therapy.

Notably, we found that the frequency of memory CD4 T cells that produce IL-10 upon mitogenic stimulation is transiently increased after the first dose of natalizumab. It is tempting, therefore, to speculate that natalizumab may skew the CD4 T cell response toward IL-10 production and away from production of IFNγ, TNF, and IL-2. This suggests a possible mechanism by which natalizumab treatment could lead to PML, as 50% of the subjects with natalizumab-associated PML that we studied produced IL-10 in response to JCV, and the other 50% had no measurable T cell response to the virus. There are a number of mechanisms by which natalizumab treatment could potentially skew the CD4 T cell response toward IL-10 production, including increasing antigen load through mobilization of infected CD34^+^ cells [8], which may affect the cytokine profile [32], altering the antigen-presenting cells (APCs) that interact with the T cells [25], or altering T cell trafficking and thus with which APCs the T cells interact [5]–[7]. There is also the possibility that a direct effect of natalizumab on T cells could affect cytokine production, as may occur with ribavirin treatment for hepatitis C virus [33].

The low probability that any of the natalizumab-treated MS study subjects could go on to develop PML is consistent with the lack of JCV-specific IL-10 production in those subjects who did not have PML. Furthermore, in this study we were only able to measure T cell responses in the peripheral blood and were not able to sample the CNS of subjects without PML. A previous study showed that after 12 months of natalizumab treatment, levels of IL-10 mRNA were increased in bulk CSF cells, while remaining unchanged in PBMC [19]. Although this finding may be due to altered CSF cell subset composition after treatment rather than upregulation of IL-10, it supports the hypothesis that natalizumab may alter the cytokine milieu in the CNS. Indeed, we found increased levels of IL-10 and IL-5 in CSF samples from individuals with natalizumab-associated PML. Although these cytokines are not typically associated with control of virus replication or with each other, their aberrant production may be indicative of an immune response that fails to control JCV replication at the site of disease. The increased CSF IL-15 we observed early in PML disease is consistent with inflammatory CNS disease [34]–[37] but is unlikely to be due solely to MS disease-associated inflammation [36]–[38] as the PML and non-PML groups both consisted of individuals with MS who were treated with natalizumab and had neurological symptoms consistent with PML. Thus far, it is not possible to demonstrate a causative link between IL-10 production and PML as the very low incidence of natalizumab-associated PML makes a prospective study unlikely. However, we believe that the potential mechanism suggested by these data should inform future work.

Taken together, our data provide a framework for understanding immune control of JC viremia and the development of PML and suggest avenues of investigation to allow the better monitoring, treatment and prevention of PML in natalizumab-treated people. First, our finding that subjects with PML lacked JCV-specific T cell responses or produced IL-10 in response to stimulation suggests that immune monitoring might identify natalizumab-treated individuals who are at risk of developing PML, by screening subjects prior to treatment or while on treatment. None of the MS patients without PML or healthy subjects included in our study showed an absent or IL-10 producing T cell response similar to that observed in the subjects with PML, and this suggests that individuals with these phenotypes are relatively rare and could be identified by immune monitoring prior to treatment. The potential of such screening of JCV-specific T cell responses to identify a small number of individuals at risk for the development of PML could be complementary to stratification strategies based on antibody levels that are currently being tested to identify approximately 50% of treated individuals who are at increased risk [2], [39]. Second, the unique IL-10 response to JCV in two PML cases and the increased levels of IL-10 in the CSF of subjects with PML suggests that IL-10 or the IL-10 receptor may be potential therapeutic targets in natalizumab-associated PML [26]. Finally, the poor magnitude or quality of the memory T cell response to JCV in subjects with PML suggests that a vaccine which boosts JCV-specific T cells that produce IFNγ, TNF and IL-2 could play a role in the prevention of natalizumab-associated PML.

## Materials and Methods

### Ethics statement

The study was approved by the IRB of the University of Texas Southwestern Medical Center. Written informed consent was obtained from all study subjects.

#### Study subjects

Eight individuals were recruited who were initiating natalizumab therapy (Tysabri, Biogen Idec) at the University of Texas Southwestern Medical Center Multiple Sclerosis Clinic. Subjects underwent a washout period of at least two weeks and had blood samples taken immediately before the first dose and each subsequent monthly infusion. Ten healthy blood donors were recruited at the NIH Vaccine Research Clinic. Blood samples from 4 subjects with natalizumab-associated PML were obtained, and CSF samples from these individuals and 6 others, as well as 10 control subjects who had suspected PML but a negative test for JCV were supplied by the NIH Laboratory of Molecular Medicine and Neuroscience clinical testing lab. Tables S1, S2, S3 describe certain clinical and laboratory characteristics of all the included natalizumab-treated patients.

### Cell isolation

Peripheral blood mononuclear cells (PBMC) were isolated from whole blood by ficoll-hypaque density centrigugation (GE Heathcare Life Sciences). PBMC were cryopreserved in freezing media containing 90% fetal bovine serum and 10% DMSO for use in T cell assays.

### Cell stimulation

Frozen PBMC were thawed and washed twice with RPMI 1640 supplemented with 10% heat inactivated fetal calf serum, 100 U/ml penicillin G, 100 U/ml streptomycin sulfate, and 1.7 mm sodium glutamine (R-10) containing 10 U/ml DNase I (Roche Diagnostics). Cells were then rested for two hours before being washed and then plated in 96-well plates in 200 uL final volume of R10 with DNase I. All experiments were done at 5×10^6^ PBMC/ml in the presence of 1 µg/ml each of αCD28 and αCD49d (BD Bioscience), in the absence or presence of peptide antigens or SEB. Cells were stimulated for 6 hours, with 10 µg/ml brefeldin A (BFA) (Sigma Chemical Company) added after 1 hour. For subjects PML-1, PML-2 and PML-3, freshly isolated PBMC were stimulated for 16 hours with BFA added after 1 hour. For subject PML-4 frozen PBMC were stimulated for 6 hours as described in parallel with MS and healthy subjects.

### Antibodies

Directly conjugated monoclonal antibodies (mAbs) specific for the molecules listed were obtained from the following: CD3 APC-H7, TNF Cy7PE, IFNγ V450, IL-10 APC, IL-10 PE, IL-2 FITC, CD3 Alexa700, IFNγ FITC, IL-2 APC from BD Biosciences; CD45RO-TRPE, CD27-Cy5PE from Beckman Coulter; CD4-Cy55PE from Caltag, CD8 QD705 from Invitrogen, and IL-2 Alexa700 from BioLegend. The following antibodies were conjugated in our laboratory according to standard protocols (http://drmr.com/abcon/index.html): CD57-QD585, CD14-QD605, CD14 PacificBlue and CD19-QD655.

### Immunofluorescence staining

Stimulated PBMC used for intracellular cytokine staining were washed and pre-stained for 10 minutes with a pre-titered amount of LIVE/DEAD fixable aqua dead cell stain (Molecular Probes). Cells were then surface stained with a mixture of pre-titered amounts of directly conjugated antibodies to CD27, CD45RO, CD57, CD4, CD8, CD19, and CD14 made to a total volume 100 µl with Delbecco's phosphate buffered saline (PBS). Cells were stained for 30 min at 4°C in the dark. Cells were then washed and permeabilized using the cytofix/cytoperm kit (BD Biosciences) according to the manufacturer's instructions. After intracellular staining for CD3, IFNγ, TNF, IL-2 and IL-10 cells were washed one final time and fixed in PBS containing 1% paraformaldehyde and then stored at 4°C. Flow cytometric analysis was done within 24 h of staining.

### Flow cytometric analysis

Cells were analyzed with a modified LSRII (BD Immunocytometry Systems) equipped for the detection of 18 fluorescence parameters. Between 500,000 and 1,000,000 events were collected for each sample. Electronic compensation was conducted with antibody capture beads (BD Biosciences) stained separately with individual mAbs used in test samples. All analytical gating was performed using FlowJo version 9.0.1 (Tree Star, Inc. Ashland, OR).

### Gating

CD4 and CD8 memory T cells were identified by sequential gating, using the same gating scheme for all analyzed samples. Cells were identified as lymphocytes by Side Scatter Area (SSC-A) and Forward Scatter Area (FSC-A), and as singlets by Forward Scatter Area (FSC-A) and Forward Scatter Height (FSC-H). Memory CD4 and CD8 cells were defined as Aqua LIVE/DEAD stain^−^, CD14^−^, CD19^−^, CD3^+^, CD4^+^ and CD8^−^ or CD8^+^ and CD4^−^, and memory cells were defined as CD45RO^+^ or CD45RO^−^ and CD27^−^. Cells positive for IFNγ, TNF, IL-2 and IL-10 were expressed as a percentage of either memory CD4 or memory CD8 T cells. In all samples other than that from subject PML-3, IL-10^+^ cells were defined as cells that were positive for both anti-IL-10 PE and anti-IL-10 APC. In PML-3, IL-10^+^ cells were defined by anti-IL-10 PE alone.

### Peptide antigens

An amino acid sequence for the entire JCV coding region was constructed based on Mad1 that also included any common variants from NCBI and the literature [40], in order to cover the vast majority of variation in the viral epitopes that are presented *in vivo* in our patient population (particularly focusing on genotypes 1, 3, 4 and 6). 15mers overlapping by 11 amino acids were generated from this sequence, to create 381 peptides, plus 162 additional peptides containing variant amino acids, for a total of 543 peptides. These were dissolved in DMSO and pooled to create 5 peptide pools: the VP1, large T antigen and agnoprotein pools contained the entire sequence of these proteins, while the VP2 pool contained only peptides that were not shared with VP1, and the small t antigen pool contained peptides not shared with large T plus 5 additional peptides that cover the T′ splice variants [41]. The number of peptides in each pool was Agnoprotein – 27, VP1 – 149, VP2 – 109, large T – 222, small t – 36. Because the peptide stimulations were done in pools rather than individually, and contained multiple variants of the same peptide, it is theoretically possible that competition amongst peptides in the same pool for the same MHC protein decreased sensitivity of the assay to detect a response. Because we use high concentrations of each peptide (2 µg/ml each), this should only occur if a pool contains a peptide with at least 100-fold higher affinity for MHC than typical peptides, and thus should be a very rare event. Limitations on the number of cells available from samples prevents the assessment of all peptides individually and necessitates the commonly-used pooling approach. Peptides were obtained from New England Peptide, and were >70% pure. For measurement of CMV-specific T cells, 138 15mers overlapping by 11 amino acids corresponding to the entire CMV pp65 protein sequence were pooled and dissolved in DMSO. CMV pp65 peptides were obtained from JPT peptide technology, and were >70% pure. In pooled peptide mixes, each peptide was at a concentration of 400 µg/ml. Five µl were added for each ml of assay volume. Final concentration of peptides was 2 µg/ml.

#### Plasma and CSF cytokines

Cytokines were measured using the Bio-Plex Pro Human Cytokine 27-plex Assay according to manufacturer's instructions (Bio-Rad Laboratories, Hercules, CA). CSF samples were assayed at a dilution of 1∶2 due to sample volume limitations.

### Statistical analysis

Statistical comparisons were performed using Prism (GraphPad Software, San Diego, CA). Experimental variables were analyzed using Fisher's Exact test, Mann-Whitney U test or Wilcoxon matched-pairs signed rank test. Bars depict median values. P-values <0.05 were considered significant. Analysis and graphical representation of cytokine production was conducted by using the data analysis program Simplified Presentation of Incredibly Complex Evaluations (SPICE version 5.05013 Beta) [28].

## Supporting Information

Figure S1 **JCV-specific memory T cell responses in healthy subjects.** CD4 (top) and CD8 (bottom) memory T cell responses from all ten healthy subjects are shown, with the background-subtracted magnitude of the response to each JCV protein depicted by colored bars. Responses were measured by production of any combination of IFNγ, TNF and IL-2. The finding that all non-PML subjects had T cells that responded to JCV peptide pools does not necessarily indicate they were infected with JCV. It is possible that such responses may be specific for BK virus, as it shares significant homology with JCV. As negative serology does not definitively rule out latent JCV infection one cannot fully distinguish JCV-infected from uninfected subjects cross-sectionally. However, the presence of T cells that respond to JCV, whether or not these cells were originally primed to JCV itself, has implications for the control of new infection or reactivation of latent JCV.
(PDF)

Figure S2 **JCV-specific IL-10 response in subject PML-4.** PBMC were stimulated for 6 hours with costimulatory molecules alone (left) or with the addition of VP1 peptides (right). Plots show memory CD4 T cells. The X-axis shows fluorescence intensity for IL-10 PE, and the Y-axis shows fluorescence intensity for IL-10 APC. The diagonal population are IL-10^+^ cells.
(PDF)

Table S1 **Characteristics of longitudinal subjects initiating therapy with Natalizumab.** Characteristics of subjects with MS initiating therapy with natalizumab are shown, including subject number, age, gender, diagnosis and estimated Expanded Disability Status Scale (EDSS).
(DOCX)

Table S2 **Characteristics of subjects with Natalizumab-associated PML analyzed for T cell responses.** For the subjects with natalizumab-associated PML included in the T cell response analysis, the CSF viremia at time of diagnosis is listed, along with the plasma viremia at the time of sampling, the number of doses of natalizumab that the subject received, and the length of time from diagnosis to sampling for the T cell assays.
(DOCX)

Table S3 **Characteristics of subjects with Natalizumab-associated PML who were analyzed for CSF cytokine levels.** For the subjects with natalizumab-associated PML included in the CSF cytokine analysis, the CSF viral load at diagnosis (timepoint 1) and at a later timepoint (timepoint 2) are shown, with the length of time between the diagnostic sample (timepoint 1) and the later sample (timepoint 2).
(DOCX)
