# Supplementary figures and images for: Changes in JC Virus-Specific T Cell Responses during Natalizumab Treatment and in Natalizumab-Associated Progressive Multifocal Leukoencephalopathy
Source: PLoS Pathog. 2012 Nov 8;8(11):e1003014. doi: 10.1371/journal.ppat.1003014 (PMC3493478; doi:10.1371/journal.ppat.1003014)

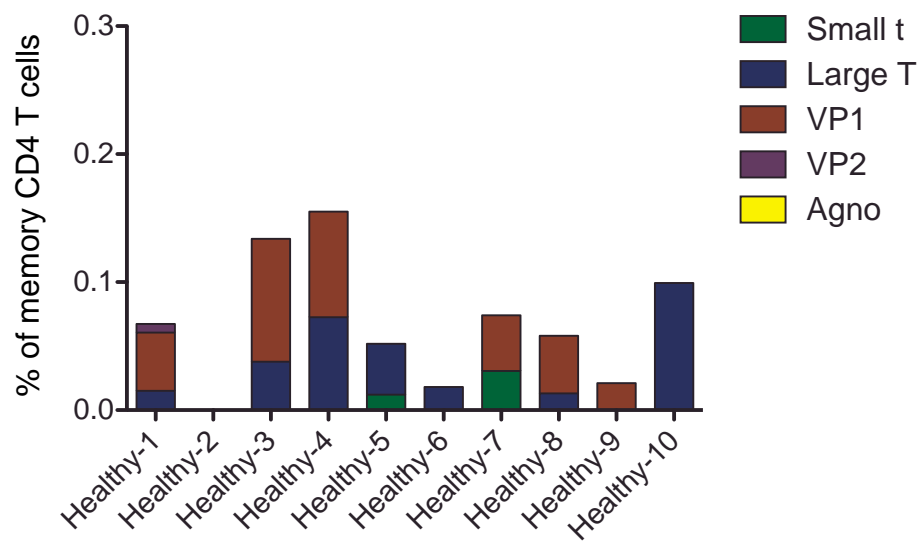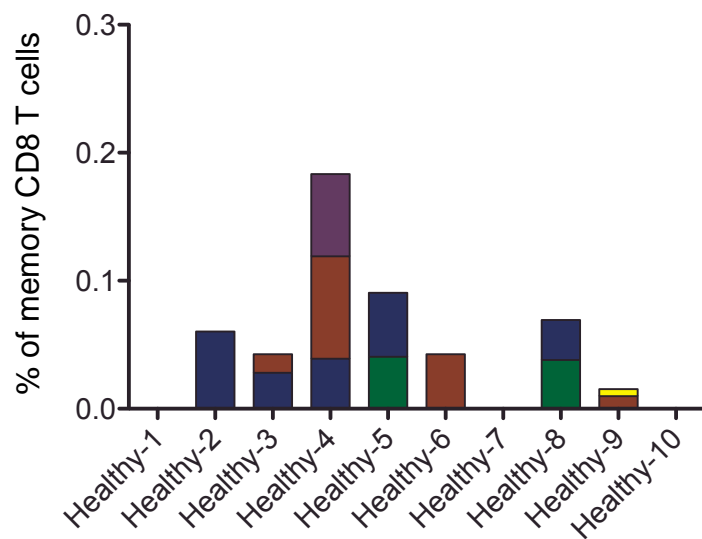

Perkins Supplementary Figure 1

Supplement: Figure S1 — JCV-specific memory T cell responses in healthy subjects. CD4 (top) and CD8 (bottom) memory T cell responses from all ten healthy subjects are shown, with the background-subtracted magnitude of the response to each JCV protein depicted by colored bars. Responses were measured by production of any combination of IFNγ, TNF and IL-2. The finding that all non-PML subjects had T cells that responded to JCV peptide pools does not necessarily indicate they were infected with JCV. It is possible that such responses may be specific for BK virus, as it shares significant homology with JCV. As negative serology does not definitively rule out latent JCV infection one cannot fully distinguish JCV-infected from uninfected subjects cross-sectionally. However, the presence of T cells that respond to JCV, whether or not these cells were originally primed to JCV itself, has implications for the control of new infection or reactivation of latent JCV. (PDF) [file ppat.1003014.s001.pdf]

Control

VP1

IL-10 APC

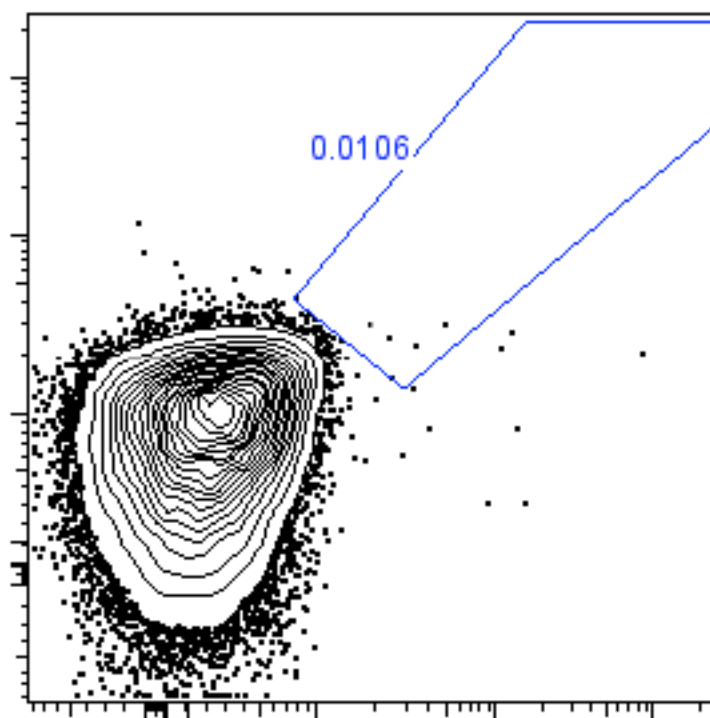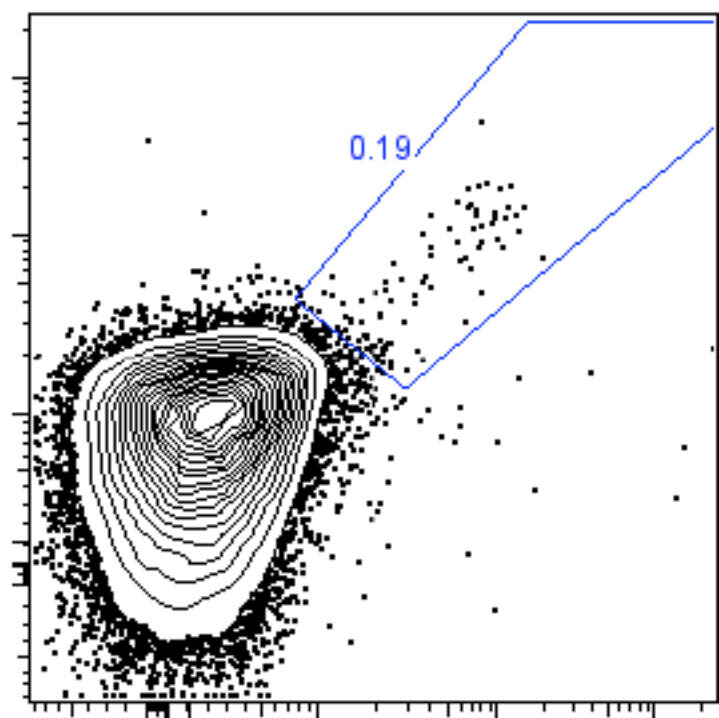

IL-10 PE

Supplement: Figure S2 — JCV-specific IL-10 response in subject PML-4. PBMC were stimulated for 6 hours with costimulatory molecules alone (left) or with the addition of VP1 peptides (right). Plots show memory CD4 T cells. The X-axis shows fluorescence intensity for IL-10 PE, and the Y-axis shows fluorescence intensity for IL-10 APC. The diagonal population are IL-10+ cells. (PDF) [file ppat.1003014.s002.pdf]
