# Supplementary material for: Changes in JC Virus-Specific T Cell Responses during Natalizumab Treatment and in Natalizumab-Associated Progressive Multifocal Leukoencephalopathy
Source: PLoS Pathog. 2012 Nov 8;8(11):e1003014. doi: 10.1371/journal.ppat.1003014 (PMC3493478; doi:10.1371/journal.ppat.1003014)
Supplement: Table S1 — Characteristics of longitudinal subjects initiating therapy with Natalizumab. Characteristics of subjects with MS initiating therapy with natalizumab are shown, including subject number, age, gender, diagnosis and estimated Expanded Disability Status Scale (EDSS). (DOCX) [file ppat.1003014.s003.docx]

**Table S1: Characteristics of Longitudinal Subjects Initiating Therapy with Natalizumab**

| Subject | Age | Gender | Diagnosis | Estimated EDSS |
| --- | --- | --- | --- | --- |
| MS-009 | 26 | M | RRMS | <4 |
| MS-010 | 40 | F | SPMS | 8.5 |
| MS-012 | 66 | F | RRMS/SPMS | 6.5 |
| MS-013 | 42 | F | RRMS | <5 |
| MS-016 | 38 | F | RRMS | <5 |
| MS-018 | 58 | F | RRMS | 7.5 |
| MS-021 | 64 | F | RRMS | <5.5 |
| MS-025 | 42 | M | RRMS | <5.5 |
