# Supplementary material for: Changes in JC Virus-Specific T Cell Responses during Natalizumab Treatment and in Natalizumab-Associated Progressive Multifocal Leukoencephalopathy
Source: PLoS Pathog. 2012 Nov 8;8(11):e1003014. doi: 10.1371/journal.ppat.1003014 (PMC3493478; doi:10.1371/journal.ppat.1003014)
Supplement: Table S2 — Characteristics of subjects with Natalizumab-associated PML analyzed for T cell responses. For the subjects with natalizumab-associated PML included in the T cell response analysis, the CSF viremia at time of diagnosis is listed, along with the plasma viremia at the time of sampling, the number of doses of natalizumab that the subject received, and the length of time from diagnosis to sampling for the T cell assays. (DOCX) [file ppat.1003014.s004.docx]

**Table S2: Characteristics of Subjects with Natalizumab-associated PML Analyzed for T Cell Responses**

| Subject | CSF Viremia at diagnosis (copies/mL) | Plasma viremia at sampling (copies/mL) | Doses of natalizumab | Time from diagnosis to sampling |
| --- | --- | --- | --- | --- |
| PML-1 | 21736 | 63 | 30 | 2 weeks |
| PML-2 | 8925 | 31 | 22 | 2 months |
| PML-3 | 39 | 1277 | 24 | 4 months |
| PML-4 | 6050 | 16 | 28 | 5 years |
