# Supplementary material for: Changes in JC Virus-Specific T Cell Responses during Natalizumab Treatment and in Natalizumab-Associated Progressive Multifocal Leukoencephalopathy
Source: PLoS Pathog. 2012 Nov 8;8(11):e1003014. doi: 10.1371/journal.ppat.1003014 (PMC3493478; doi:10.1371/journal.ppat.1003014)
Supplement: Table S3 — Characteristics of subjects with Natalizumab-associated PML who were analyzed for CSF cytokine levels. For the subjects with natalizumab-associated PML included in the CSF cytokine analysis, the CSF viral load at diagnosis (timepoint 1) and at a later timepoint (timepoint 2) are shown, with the length of time between the diagnostic sample (timepoint 1) and the later sample (timepoint 2). (DOCX) [file ppat.1003014.s005.docx]

**Table S3: Characteristics of Subjects with Natalizumab-associated PML Who Were Analyzed for CSF Cytokine Levels**

| Subject | CSF VL timepoint 1 | CSF VL timepoint 2 | time between samples |
| --- | --- | --- | --- |
| PML-1 | 21736 | 1070 | 2 months |
| PML-2 | 8925 | 613* | 2 months* |
| PML-3 | 1081 | no sample | no 2nd sample |
| PML-4 | 2275 | undetected | 4 years |
| PML-5 | 3879 | 82 | 3 months |
| PML-6 | 215 | 26 | 5 months |
| PML-7 | 96436 | 1114 | 5 months |
| PML-8 | 2374 | undetected | 7 months |
| PML-9 | 155 | undetected | 8 months |
| PML-10 | 51 | undetected | 4 months |

*CSF timepoint 2 sample for subject PML-2 was not available at time of cytokine testing, and was not included in CSF cytokine analysis.
